# Ipsilateral Vestibular Schwannoma after Cochlear Implantation

**DOI:** 10.1155/2022/4918785

**Published:** 2022-02-18

**Authors:** S. Tüpker, N. Ay, L. U. Scholtz, H. B. Gehl, V. F. Mautner, P. Goon, H. Sudhoff, I. Todt

**Affiliations:** ^1^Department of Otolaryngology, Head and Neck Surgery, Medical School OWL, Bielefeld University, Klinikum Bielefeld Mitte, Bielefeld, Germany; ^2^Department of Radiology, Medical School OWL, Bielefeld University, Klinikum Bielefeld Mitte, Bielefeld, Germany; ^3^Universitätsklinikum Hamburg Eppendorf, Neurofibromatose Ambulanz, Hamburg, Germany

## Abstract

**Objective:**

The vestibular schwannoma incidence rate is approximately 4.2 per 100,000/year. Thus far, about 700,000 cochlear implantations have been performed worldwide; therefore, the occurrence of vestibular schwannoma postcochlear implantations can be assumed to be infrequent. Recent developments allow safe observation and surveillance of the implanted-side internal auditory canal (IAC) and cochlea by magnetic resonance imaging (MRI), even after cochlear implantation. *Patients*. A 71-year-old woman with sudden hearing loss and a contralateral vestibular schwannoma without clinical and genetic signs of neurofibromatosis type II. *Intervention(s)*. Ipsilateral cochlear implantation and contralateral vestibular schwannoma extirpation with regular tumor follow-up. *Main Outcome Measure(s)*. Comparison of ipsilateral pre and postcochlear implantation 3T MRI T1 GAD.

**Results:**

We observed a tumor growing at the fundus of the internal auditory canal 1 year after cochlear implantation on the ipsilateral side. Although first detected after cochlear implantation beside a known vestibular schwannoma on the contralateral side, a scan slice thickness of 2 mm cannot fully exclude the preoperative persistence of a small tumor. Based on the clinical findings and after genetic exclusion of NFII, the patient was classified as a NFII mosaic type.

**Conclusion:**

Even after cochlear implantation, tumors in the IAC causing vertigo, facial palsy, and affecting the audiologic outcome can be detected by MRI. The MRI slice thickness used before cochlear implantation should be under 2 mm.

## 1. Introduction

The occurrence of acoustic neuroma is described to be up to 4.2 per 100,000/y. A rising incidence has been detected due to the existence of better diagnostic tools and better access to healthcare [1, 2]. With the current number of cochlear implantees being about 700,000 globally, the probability of new occurrence of an acoustic neuroma after cochlear implantation is low but not negligible. High-resolution computed tomography is often unable to identify vestibular schwannoma; thus, magnetic resonance imaging (MRI) usually is performed as part of the regular preoperative evaluation to exclude this kind of tumor [3, 4].

The MRI scan slice thickness was previously set at 2 mm. Therefore, besides the occurrence of new acoustic neuroma after cochlear implantation, a limited probability exists of undetected small schwannomas being present before cochlear implantation. The development of cochlear implant magnets and increasing knowledge of implant and head position make it possible to perform MRI after cochlear implantation for postoperative visual assessment of the cochlea and internal auditory canal. For example, an evaluation of vestibular schwannoma and intralabyrinthine schwannoma after cochlear implantation was shown to be successful [5].

## 2. Case Report

To the best of our knowledge, we are presenting the first case of a cochlear implantee with an ipsilateral vestibular schwannoma, detected 2 years after initial cochlear implantation.

**Figure 1:**
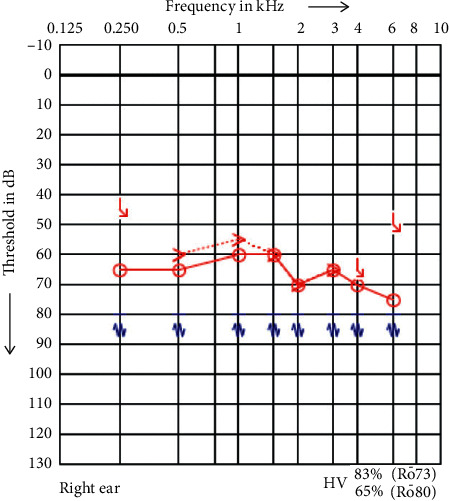
PTA of the right side 2014.

In 2014, the patient (a 72-year-old woman) visited our clinic for the first time and had suffered for 15 years from known intracanalicular vestibular schwannoma with deafness on the left side and moderate hearing loss of 60–70 dB on the right side (Figure 1). The patient used a hearing aid for the right-sided hearing loss. The vestibular schwannoma was constant in size and observed following a watch-and-scan strategy. She never suffered from vertigo. cVEMP testing was not performed. Clinical observation showed no signs of neurofibromatosis type II (NFII). Additional vestibular schwannoma, astrozytome, intracranial calcification, meningioma, cutaneous neurofibroma, and subcapsular cataracts were not observed.

**Figure 2:**
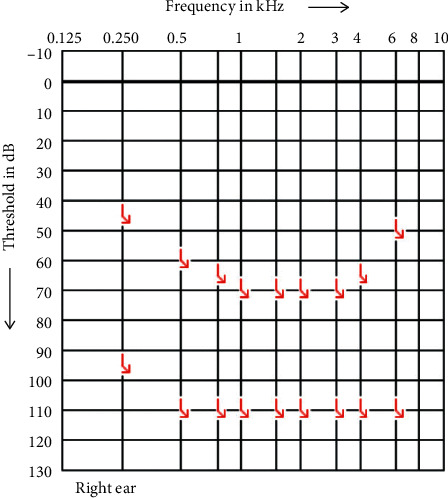
PTA of the right side after sudden hearing loss 2018.

**Figure 3:**
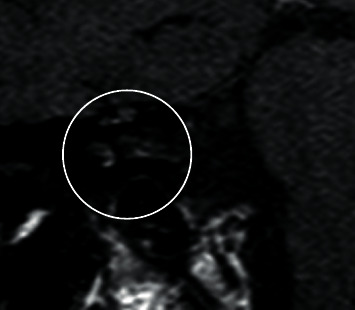
Coronal T1 MRI GAD of cerebellopontine angle before cochlear implantation of the right ear.

Sudden complete hearing loss on the right side occurred in 2018 (Figure 2), and a cochlear implantation (Synchrony® MED-EL, Innsbruck, Austria) was performed on the ipsilateral side to preserve the patient's ability to hear. The preoperative MRI T1 (gadolinium) GAD slice thickness scan was 2 mm. No vestibular schwannoma was found on the right side (Figure 3).

The patient showed an audiological performance after cochlear implantation with a monosyllabic understanding in quite of 65% at 65 dB after 6 months. After reaching a stable audiological result on the right side, promontory testing on the left side was performed and a positive response was observed. Consequently, translabyrinthine resection of the left vestibular schwannoma was performed.

**Figure 4:**
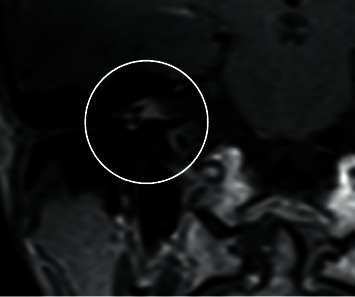
Coronal T1 MRI GAD of cerebellopontine angle 2 years after cochlear implantation of the right ear.

**Figure 5:**
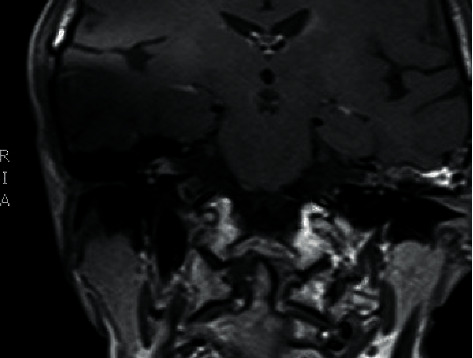
Coronal overview T1 MRI GAD of cerebellopontine angle 2 years after cochlear implantation of the right ear.

**Figure 6:**
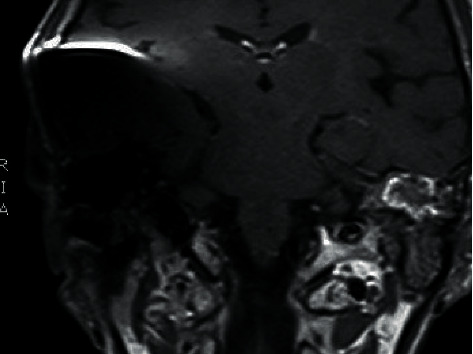
Coronal overview T1 MRI GAD of cerebellopontine angle 2 years after cochlear implantation of the right ear with slices behind the cochlea indicating local artifact size.

During her regular check-up with MRI control 1 year after surgery, the T1 GAD-based sequence raised suspicion of vestibular schwannoma on the right side (Figures 4 and 5).  Figure 6 shows that the patient underlines the importance of the right handling of MRI artifact by showing the maximum extent of MRI artifact. The audiological outcome was stable with a monosyllabic speech understanding of 65% at 65 dB. Currently, a watch-and-scan strategy is performed for the right side. Further options in case of growth (e.g., cyber/gamma knife radiation) are discussed.

Based on the clinical pattern of a bilateral schwannoma, the patient was classified as a case of neurofibromatosis type II (NFII).

NFII is a hereditary disease (Merlin coding gene) characterized by benign symmetrical tumors of the IAC in 90% of the cases.

Sanger sequencing and multiplex ligation-dependent probe amplification (MLPA) did not reveal NFII genetic alterations in blood or tumor specimen. There was no suspicion of NFII beforehand, and there had been no other symptoms nor signs of NFII until that time point. The lack of clinical signs and symptoms in this case, therefore, raised suspicion of a case of mosaic NFII. Mosaic cases of NFII have mild clinical progression and are caused by missense mutations. A postoperative promontory testing of the left side is planned.

## 3. Discussion

The present case shows the importance of conducting MRI in cases of sudden hearing loss and contralateral known vestibular schwannoma. This finding underlines the importance of performing MRI scans even in cases of asymmetric hearing loss.

The new developments in cochlear implant magnets, implant position, and head position inside the scanner make the follow-up possible, even for cochlear implantees [5].

In the present case, although the radiological finding in the IAC was observed 2 years after the initial cochlear implantation, it remains unclear if the finding was present preoperatively but was undetected due to the slice thickness of 2 mm or if we observed a newly occurring tumor.

A 2 or 3 mm slice thickness was the preoperative standard in our clinic. This standard might be insufficient, as this case possibly shows. A thinner slice thickness of under 2 mm might solve the problem of small, so far undetected VS. We changed our standard to 0.6 mm. The second explanation for this case is the new occurrence of a vestibular schwannoma after cochlear implantation. Given the rate of 4.2 per 100,000/y, the new occurrence of such a case is rare but possible.

Additionally, other tumors such as lipoma or hemangioma can occur and radiologically mimic schwannoma [6]. Cases of NFII occurring with a cochlear implant are well described, and the procedure was performed to stabilize hearing capacity [7–9]. In this case, the NFII diagnosis was based on the clinical observation of bilateral schwannoma. Absence of characteristic clinical findings and negative somatic DNA findings make this diagnosis controversial. An alternative diagnosis as an NFII mosaic case would be a possibility. Mosaic cases are characterized by mild clinical progression and missing or missense mutation as a genetic finding.

Apart from this specific case, a new occurrence of sporadic vestibular schwannoma must be considered in cases of reduction of postoperative speech perception after cochlear implantation. Even the new occurrence of vertigo and facial palsy after cochlear implantation could be caused by postimplantation changes inside the IAC or labyrinthine. In cases of deafness with a contralateral schwannoma, an NFII case must be considered.

## 4. Conclusion

MRI observation after cochlear implantation allows for monitoring changes in the IAC. The preimplantation MRI slice thickness should be under 2 mm to decrease the risk of small undetected VS. Vestibular schwannoma occurrence after cochlear implantation should be considered in cases of performance loss, vertigo, facial palsy, and contralateral schwannoma.

## Data Availability

The data used to support the findings of this study are available from the corresponding author upon request.

## References

[1] Marinelli J. P., Lohse C. M., Carlson M. L. (2018). Incidence of vestibular schwannoma over the past half-century: a population-based study of Olmsted County, Minnesota. *Otolaryngology-Head and Neck Surgery*.

[2] Kshettry V. R., Hsieh J. K., Ostrom Q. T., Kruchko C., Barnholtz-Sloan J. S. (2015). Incidence of vestibular schwannomas in the United States. *Journal of Neuro-Oncology*.

[3] Yigit O., Kalaycik Ertugay C., Yasak A. G., Araz Server E. (2019). Which imaging modality in cochlear implant candidates?. *European Archives of Oto-Rhino-Laryngology*.

[4] Tamplen M., Schwalje A., Lustig L., Alemi A. S., Miller M. E. (2016). Utility of preoperative computed tomography and magnetic resonance imaging in adult and pediatric cochlear implant candidates. *The Laryngoscope*.

[5] Sudhoff H., Gehl H. B., Scholtz L. U., Todt I. (2020). MRI observation after intralabyrinthine and vestibular schwannoma resection and cochlear implantation. *Frontiers in Neurology*.

[6] Dazert S., Aletsee C., Brors D. (2005). Rare tumors of the internal auditory canal. *European Archives of Oto-Rhino-Laryngology*.

[7] Nölle C., Todt I., Basta D., Unterberg A., Mautner V. F., Ernst A. (2003). Cochlear implantation after acoustic tumour resection in neurofibromatosis type 2: impact of intra- and postoperative neural response telemetry monitoring. *ORL*.

[8] Carlson M. L., Breen J. T., Driscoll C. L. (2012). Cochlear implantation in patients with neurofibromatosis type 2. *Otology & Neurotology*.

[9] Lloyd S. K. W., King A. T., Rutherford S. A. (2017). Hearing optimisation in neurofibromatosis type 2: a systematic review. *Clinical Otolaryngology*.

